# Placental and maternal microbiome adaptations in a preeclamptic-like mouse model

**DOI:** 10.3389/frmbi.2026.1881824

**Published:** 2026-07-29

**Authors:** Kalie F. Beckers, Christopher J. Schulz, Juliet P. Flanagan, Chin-Chi Liu, Gary W. Childers, Isabella N. Faulkner, Jenny L. Sones

**Affiliations:** 1 Division of Veterinary Medicine, Tulane National Primate Research Center, Tulane University, Covington, LA, United States; 2 Veterinary Clinical Sciences, School of Veterinary Medicine, Louisiana State University, Baton Rouge, LA, United States; 3 Department of Biological Sciences, Southeastern Louisiana University, Hammond, LA, United States; 4 Office of Research and Graduate Education, School of Veterinary Medicine, Louisiana State University, Baton Rouge, LA, United States; 5 Clinical Sciences, Colorado State University College of Veterinary Medicine and Biomedical Sciences, Fort Collins, CO, United States

**Keywords:** maternal microbiome, obesity, placenta microbiome, preeclampsia, pregnancy

## Abstract

**Introduction:**

The maternal microbiome plays a crucial role in pregnancy with growing evidence supporting vertical microbial transmission from mother to fetus. The placenta, once considered sterile, may serve as a conduit for this transfer. We hypothesized that the placental microbial signatures would be distinctly different from oral, fecal, or vaginal microbiomes in pregnant mice.

**Methods:**

To test this hypothesis, the obese BPH/5 mouse (n=15), which spontaneously develops a preeclampsia (PE)-like phenotype, was compared to normotensive C57 (n=8) pregnant mice. 16S rRNA gene sequencing and bioinformatic analyses were conducted to assess microbial diversity and composition from samples collected at embryonic day 18.5 (feces, oral cavity, vagina, and placenta).

**Results:**

Alpha diversity analysis revealed that oral microbiomes of both BPH/5 and C57 were significantly less diverse compared to the placental microbial signatures (p = 0.019 and <0.001, respectively). Beta diversity analysis confirmed distinct microbial communities across body sites and between strains (p < 0.001), while no significant differences were detected in placental and vaginal microbiomes (p > 0.05). Microbial composition analysis showed site-specific variations at the phylum and genus levels with Firmicutes and Bacteroidetes being dominant across all sites. BPH/5 placentas were enriched in *Alistipes*, *Lachnospiraceae_NK4A136* and *Helicobacter*. In contrast, C57 placentas were enriched in *Alistipes*, *Lachnospiraceae_NK4A136*, and *Lactobacillus* suggesting strain-specific microbial alterations with common genera between maternal oral, fecal, vaginal, and placental communities.

**Discussion:**

These findings demonstrate that the placental microbial signatures are unique in a PE-like mouse model. Further studies are needed to elucidate the functional impact of these microbial differences on maternal and fetal PE outcomes.

## Introduction

For nearly 20 years, the role of microbiomes in mammalian life has been explored. The microbiome has been shown to be closely involved in essential physiological activities, including metabolic processes and immune function. Body site-specific microbiomes have been shown to develop early in life (Ferretti et al., 2018; Ward et al., 2018; Bogaert et al., 2023). A current hypothesis is that certain neonatal microbial colonies originate from the mother during the perinatal period (Flores Ventura et al., 2025). Transfer likely begins from the onset of pregnancy but is affected by multiple factors, such as maternal health status, diet, genetics, lifestyle, mode of delivery, and infant feeding patterns (Xiao and Zhao, 2023). Long-term studies are needed to show that bacterial translocation from mother to neonate plays a vital role in development and health throughout life.

An important connection between mother and fetus is the placenta. The placental microbiome was first characterized using next generation sequencing by Aagard et al (Aagaard et al., 2014). The once thought sterile vital organ of pregnancy may play a dynamic role in microbial fetus colonization. The placenta is responsible for the exchange of nutrients and waste products between the maternal and fetal circulatory system. It is not fully understood how bacteria get to the placenta. Many theories have been proposed such as vertical or ascending transmission from the vagina, hematogenous transfer from oral cavity, or leaky gut from the gastrointestinal (GI) tract (Galland, 1995; Perez et al., 2007; Goldenberg et al., 2008; Fardini et al., 2010; Amarasekara et al., 2015; Edwards et al., 2017). It was shown that a common oral microbe, when injected intravenously into pregnant mice, could reach the gravid uterus and cause abortion, stillbirth, and neonatal mortality (Han et al., 2004). Thus, supporting the hypothesis of vertical transfer of the maternal microbiome to the developing fetus. Previous literature in humans and horses have shown similarity between placental and oral microbiomes (Aagaard et al., 2014; Beckers et al., 2023). The theory being that hematogenous transfer occurs during pregnancy due to increased vascularization and blood flow (Han et al., 2004). There is evidence to support leaky gut syndrome, which commonly develops during pregnancy, and adverse pregnancy outcomes (Tersigni et al., 2018). Leaky gut syndrome is defined as a weakening or failure of the epithelial barrier of the GI tract caused by stress, chronic inflammation, or dysbiosis of the gut microbiome. GI barrier disruption could lead to translocation of the gut microbial components into the placenta via the blood stream (Koren et al., 2012; Beckers and Sones, 2020). Literature also demonstrates altered birth outcomes with changes in the vaginal microbiome, demonstrating the potential ascending transmission from the vagina (Goldenberg et al., 2008; Hyman et al., 2014; Witkin, 2015; Stout et al., 2017; Wee et al., 2018).

Maternal diseases in humans such as hypertension, gestational diabetes, and preeclampsia (PE) have been shown to alter the maternal microbiome (Dunlop et al., 2015). Co-morbidities such as obesity may exacerbate these changes (Edwards et al., 2017). Pre-conception obesity has been linked to maternal microbiome dysbiosis, commonly defined as a reduction in microbial diversity, and a combination of the loss of beneficial bacteria (Edwards et al., 2017). This study will investigate the maternal microbiome from multiple body sites from a mouse model that spontaneously develops PE with pre-existing obesity.

BPH/5 mice are a genetic model that spontaneously develop a PE-like phenotype during pregnancy (Davisson et al., 2002) coupled with a predisposition for obesity with increased white adipose tissue (WAT), pro-inflammatory reproductive WAT, and leptin resistance (Sutton et al., 2017). Additionally, pro-inflammatory mediators are upregulated systemically and in the placenta of BPH/5 mice (Gelber et al., 2015; Sones et al., 2016). BPH/5 offspring have low-birth weight compared to normotensive C57 control mice, indicative of fetal growth restriction (FGR). Similar to PE in women that experience perinatal mortality, litter size is severely compromised due to fetal demise (Davisson et al., 2002). Previous research demonstrated that the BPH/5 maternal fecal microbiome was altered specifically with pregnancy and differed from normotensive controls (Beckers et al., 2025). Recently, the fecal, oral and vaginal microbiome has been described in early gestation BPH/5 pregnant mice before the placenta is formed (Fan et al., 2026). We hypothesize that pregnant obese preeclamptic-like BPH/5 mice will demonstrate distinct placental microbial signatures that differs from the oral, fecal and vaginal microbiomes that are perturbed compared to lean normotensive C57 controls.

## Methods

### Animal experiments

Adult (8–12 weeks of age) female BPH/5 (RRID: MGI:7380297) (n=15 pregnant) and C57 (RRID: MGI:5652309) (n=8 pregnant) mice were used from in-house colonies in this study. C57 (C57Bl/6J) were originally sourced from Jackson Laboratories. BPH/5 mice were originally a gift from Dr. Robin Davisson, Cornell University. C57 mice have been used as control mice in previous BPH/5 studies, as they were utilized in the original eight-way cross to derive the BPH/5 strain (Davisson et al., 2002; Sones et al., 2016; Reijnders et al., 2019). Female mice were co-housed in standard cages placed in a temperature- and humidity-controlled facility, maintained on a 12-hour light/dark cycle, and fed standard mouse chow (LabDiet 5001, PMI Nutrition International, Richmond, IN) with water available ad libitum. All female mice used in this study were housed for multiple generations within the same room and were randomly chosen for timed matings. Intrastrain timed matings were performed, and detection of a copulatory plug was designated as embryonic day 0.5 (e0.5) after which the male mouse was removed, and the female mouse was single housed. All experimental protocols using animals were approved by the Louisiana State University School of Veterinary Medicine and Pennington Biomedical Research Center IACUC committee. All methods were carried out in accordance with relevant guidelines and regulations.

### Samples collected

Feces, oral swabs, vaginal swabs, and swabs of whole placentae were collected from BPH/5 and C57 pregnant dams (e18.5). To collect feces, mice were placed individually in sterile, empty cages and allowed to defecate voluntarily. To collect the placental samples, the uterus was removed *in situ* to maintain sterility during sample collection. Briefly, the whole reproductive tract was removed by incising ovarian ligaments and the vagina distal to the cervix, keeping the cervix closed, and this was placed on a separate tray. The uterus was incised adjacent to the conceptus and the whole placenta was dissected free from the fetus, and swabbed on the fetal side. All samples were placed in sterile tubes, snap frozen in liquid nitrogen, and stored at –80 °C until further analysis.

### DNA sequencing

Sample processing and sequencing were conducted with laboratory personnel blinded to strain identity. Microbial DNA was extracted from samples using the Qiagen DNeasy PowerSoil extraction kits (Qiagen, USA) according to manufacturer’s protocol. The V4 variable region of the 16S rRNA gene was amplified with PCR primers 515f/806r in a 30 cycle PCR using the DreamTaq Hot Start PCR Master Mix Kit (Thermoscieniftic, Waltham, MA). PCR was performed in 20 μl volumes and included: 2 μl (7.5 μM concentration) of forward and reverse primers, 12.5 μl of Hot Start Taq 2X Master Mix (New England BioLabs Inc., Ipswich, MA., USA), 3.5 μl of deionized water, and 2 μl of sample DNA. Thermal cycle conditions were 95˚C for 3 min for the initial denaturing step, followed by 30 cycles of 95˚C for 30 s, 50˚C for 1 min, and 72˚C for 1 min. PCR products were checked on a 2% agarose gel for correct product size formation (approx. 350 bp). Michigan State University Genomics Core performed library preparation prior to Illumina MiSeq sequencing following the manufacturer’s guidelines (Kozich et al., 2013). Reagent controls using certified DNA free water were run through library preparation and PCR and did not generate libraries. For quality control, samples submitted for sequencing included a random blank sample of technical replicates.

### Bioinformatics

Initial quality screening, demultiplexing, amplicon sequence variant (ASV) inference and chimera removal were performed using the DADA2 package (Callahan et al., 2016). ASVs were classified using the Silva Release 132 16S rRNA database (RRID: SCR_006423) (Perez et al., 2007; Quast et al., 2012; Yarza et al., 2014). Microbial community analysis (Alpha and Beta Diversity) was performed using the vegan R package (RRID: SCR_011950) (Oksanen et al., 2013). Permutational multivariate analysis of variance (PERMANOVA) (Anderson, 2005) was performed using the vegan package Adonis function, and both p-values and R² values were reported to indicate statistical significance and the proportion of variation explained. Rarefaction was not performed prior to alpha- or beta-diversity analysis. Following quality filtering, samples with fewer than 500 reads were excluded from downstream analyses. ASV counts were normalized to within-sample relative abundance by dividing each ASV count by the total read count for that sample. The resulting proportional ASV table was used for Shannon diversity, Bray–Curtis dissimilarity, PCoA, and PERMANOVA analyses. To determine differentially abundant ASVs, the ASV table was first trimmed to only include ASVs with a median abundance greater than two across all samples. Microbial source tracking was conducted using modified SourceTracker2 (Knights et al., 2011). Analyses were performed in Python 3.13.2 (RRID: SCR_008394) (Python Software Foundation, Wilmington, DE), with all scripts executed in Visual Studio Code (RRID: SCR_026031) (Microsoft Corporation, Redmond, WA). All raw sequences and metadata are available in the NCBI SRA under accession PRJNA1292389 (RRID: SCR_004891).

### Samples exclusion criteria

Seven samples yielded insufficient DNA quantity for sequencing (<200ng). Six samples were removed due to low read counts (<500). Three samples were identified as outliers, with top phylum relative abundance values greater than 1.5 times the interquartile range (IQR) above the third quartile or below the first quartile.

### Statistical methods

The power analysis was conducted using G*power 3.1.9.7 (RRID: SCR_013726). A sample size of four per group, with an alpha level of 0.05 and an effect size of 2, based on previously reported microbiome differences in the BPH/5 model, was sufficient to achieve 80% power (Beckers et al., 2025). All statistical analyses were performed using JMP Pro 18.0.1 (JMP Statistical Discovery LLC, Cary, NC) (RRID: SCR_014242). A mixed analysis of variance (ANOVA) model was used to analyze relative abundance and alpha diversity. Strain (C57 vs. BPH/5), body sites, and their interactions were included as fixed effects, and each mouse was treated as a random effect. Data that did not meet normality assumptions were logarithmically transformed prior to analysis. When significant fixed effects were detected, *post hoc* comparisons were conducted using Fisher’s protected least significant difference (LSD) test. *Post hoc* comparisons were restricted to biologically relevant strain- and body-site comparisons defined by the experimental design. Model assumptions, including normality of residuals and homoscedasticity, were verified through examination of standardized residuals and quantile plots. Significance was set at p < 0.05.

## Results

### Alpha diversity via Shannon index was significantly different between body sites and strain

**Figure 1:**
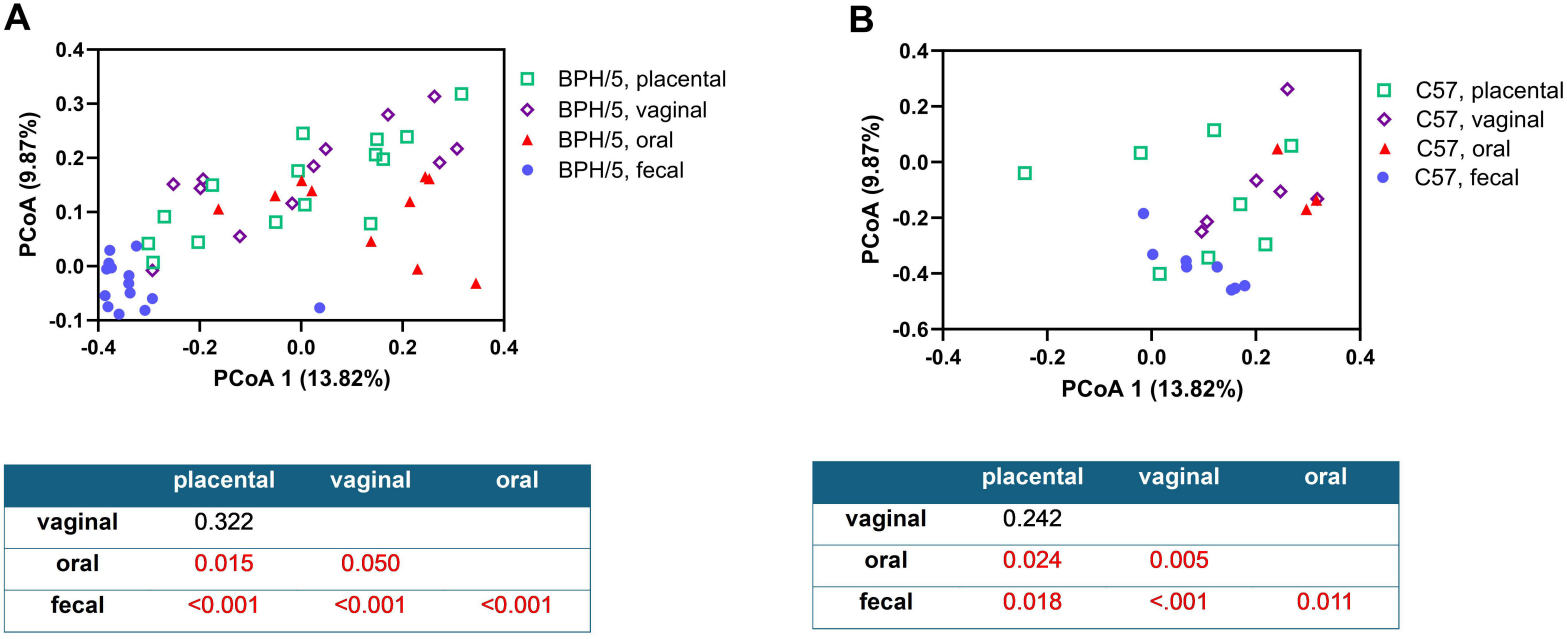
Microbial community composition differs by body site in pregnant BPH/5 **(A)** and C57 **(B)** mice using principal coordinate analysis (PCoA) of Bray–Curtis dissimilarity. Lower panels: Pairwise PERMANOVA *p*-values indicate significant differences in beta diversity between body sites within each strain. Values in red denote statistically significant differences (*p* < 0.05). Sample sizes: BPH/5: fecal (n = 14), oral (n = 10), placental (n = 15), vaginal (n = 12); C57: fecal (n = 8), oral (n = 3), placental (n = 8), vaginal (n = 6).

Of all the 85 samples processed and sequenced, five oral and one vaginal sample demonstrated low sequence reads, one oral and two vaginal samples were identified as outliers, and those nine were removed from the analysis. The remaining 76 samples presented with an overall average of 95,674 (range 660-556,059) reads from all body sites after quality filtering. Alpha diversity of the microbial communities was assessed using the Shannon diversity index (**Figure 1A**). A mixed two-way ANOVA was conducted to evaluate differences within each site and to compare each site to the placental within each strain. Within both BPH/5 and C57 strains, the oral microbiomes (BPH/5: 2.83 ± 0.26; C57: 1.09 ± 0.48) were significantly less diverse compared to the placental microbiomes (BPH/5: 3.65 ± 0.21; C57: 3.84 ± 0.29; p = 0.019 and <0.001, respectively). Due to biological variation in the oral, placental, and vaginal sites, no significant differences were observed within fecal samples (BPH/5: 4.08 ± 0.22; C57: 3.72 ± 0.29), whereas a significant difference was detected within the oral site (p = 0.002). No significant differences were observed when comparing the placental to the vaginal microbiomes (p > 0.05; BPH/5: 3.20 ± 0.24; C57: 3.47 ± 0.33).

### Bacterial community compositions were different among body sites when assessing beta diversity

**Figure 2:**
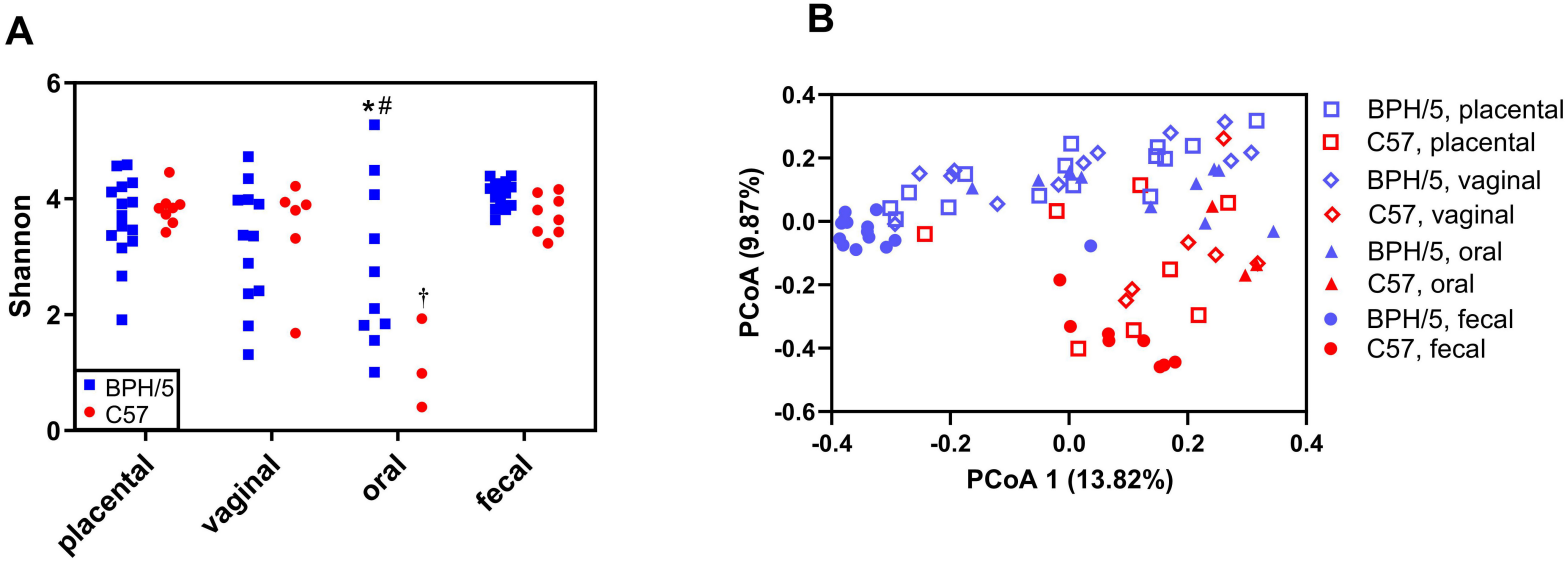
Microbial diversity in late gestation pregnant BPH/5 and C57 mice across four body sites. **(A)** Alpha diversity assessed by the Shannon diversity index significantly differed by body site and strain. Statistical analysis was performed using mixed ANOVA followed by *post hoc* pairwise LSD comparisons. (*p < 0.05 vs. C57 strain at the same site; †*p* < 0.05 vs. C57 placenta; #*p* < 0.05 vs. BPH/5 placenta). **(B)** Beta diversity analysis using Bray–Curtis dissimilarity of 16S rRNA amplicon sequence variant relative abundances revealed significant differences in microbial community composition by body site and strain (PERMANOVA, *p* < 0.001). Sample sizes: BPH/5: fecal (n = 14), oral (n = 10), placental (n = 15), vaginal (n = 12); C57: fecal (n = 8), oral (n = 3), placental (n = 8), vaginal (n = 6).

**Figure 3:**
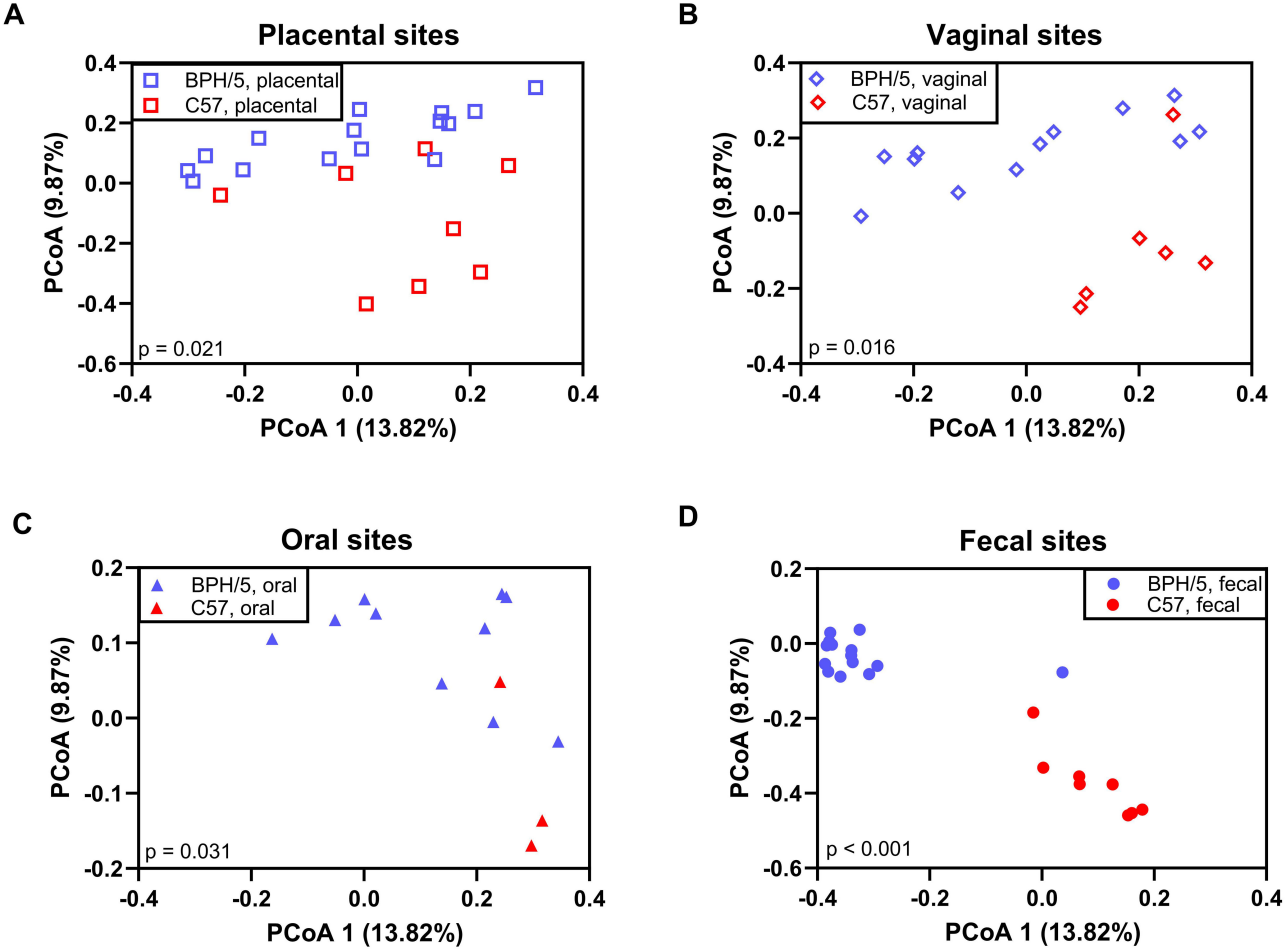
Microbial community composition differs between BPH/5 and C57 mice at individual body sites. Principal coordinate analysis (PCoA) plots based on Bray–Curtis dissimilarity of 16S rRNA amplicon sequence variant data comparing BPH/5 and C57 mice within each body site: **(A)** Placental microbiome (*p* = 0.021), **(B)** Vaginal microbiome (*p* = 0.016), **(C)** Oral microbiome (*p* = 0.031), and **(D)** Fecal microbiome (*p* < 0.001). Differences were assessed using PERMANOVA, with percent variation explained by PCoA axes indicated on the plots. Sample sizes: BPH/5: fecal (n = 14), oral (n = 10), placental (n = 15), vaginal (n = 12); C57: fecal (n = 8), oral (n = 3), placental (n = 8), vaginal (n = 6).

Microbial community composition differed significantly by body site and strain interaction when assessed using beta diversity (p < 0.001, R^2^ = 0.28, PERMANOVA with Bray–Curtis dissimilarity of 16S amplicon sequence variant relative abundances) (**Figure 1B**). In the BPH/5 model, placental microbiome was significantly different from feces (p < 0.001, R^2^ = 0.38) and the oral cavity (p=0.015, R^2^ = 0.18), but not from the vaginal microbiome (p = 0.322, R^2^ = 0.04) based on Bray–Curtis dissimilarity (**Figure 2A****).** Similarly, in C57 controls, the placental microbiome differed significantly from the fecal (p = 0.018, R^2^ = 0.17) and oral cavity (p = 0.024, R^2^ = 0.30), but not from the vaginal microbiome (p = 0.242, R^2^ = 0.09) (**Figure 2B**). When comparing each body site by strain, significant differences were observed between BPH/5 and C57 controls at the placental (p = 0.021, R^2^ = 0.14), vaginal (p = 0.016, R^2^ = 0.18), oral (p = 0.031, R^2^ = 0.20), and fecal sites (p < 0.001, R^2^ = 0.65) (**Figures 3A–D**).

### Variation in relative abundance found at phyla level among body sites

**Figure 4:**
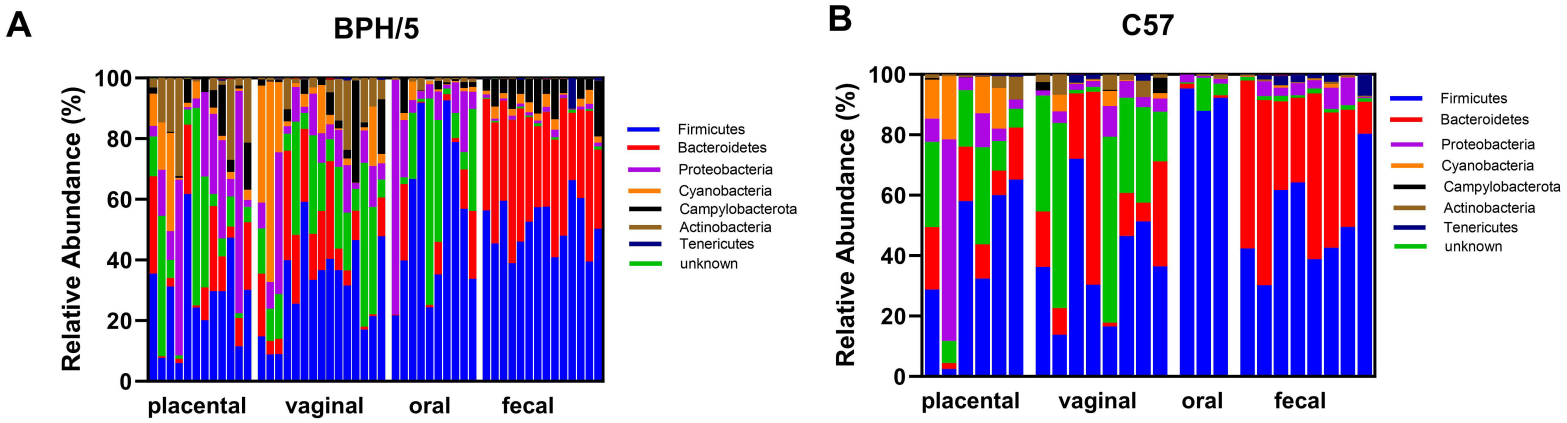
Relative abundance of dominant bacterial phyla across body sites in BPH/5 and C57 mice. Stacked bar plots show the relative abundance (%) of bacterial phyla identified in the placental, vaginal, oral, and fecal microbiota of pregnant BPH/5 **(A)** and C57 **(B)** mice. Taxonomic classification was performed at the phylum level based on 16S rRNA amplicon sequencing. Major phyla include Firmicutes, Bacteroidota, Proteobacteria, Cyanobacteria, Campylobacterota, Actinobacteria, Tenericutes, and unclassified taxa. Each bar represents an individual sample. Sample sizes: BPH/5: fecal (*n* = 14), oral (*n* = 10), placental (*n* = 15), vaginal (*n* = 12); C57: fecal (*n* = 8), oral (*n* = 3), placental (*n* = 8), vaginal (*n* = 6).

**Figure 5:**
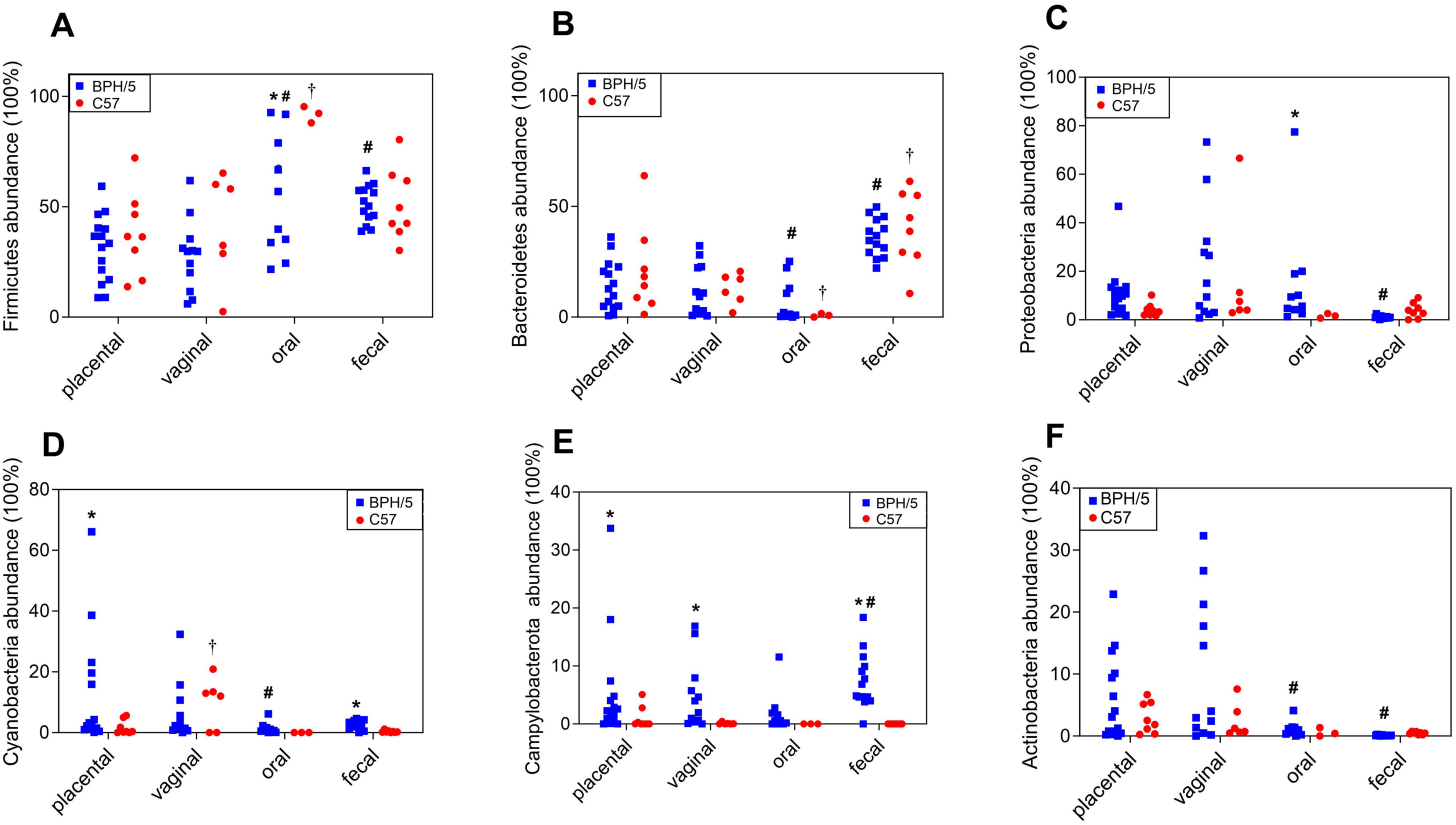
Relative abundance of bacterial phyla in BPH/5 and C57 mice across four body sites. Relative abundance of bacterial phyla in placental, vaginal, oral, and fecal samples from pregnant BPH/5 (blue squares) and C57 (red circles) mice: **(A)** Firmicutes, **(B)** Bacteroidetes, **(C)** Proteobacteria, **(D)** Cyanobacteria, **(E)** Campylobacterota, and **(F)** Actinobacteria. Statistical analysis was performed using mixed ANOVA followed by *post hoc* pairwise LSD comparisons. (*p < 0.05 vs. C57 strain at the same site; †*p* < 0.05 vs. C57 placenta; #*p* < 0.05 vs. BPH/5 placenta). Sample sizes: BPH/5: fecal (n = 14), oral (n = 10), placental (n = 15), vaginal (n = 12); C57: fecal (n = 8), oral (n = 3), placental (n = 8), vaginal (n = 6).

When evaluating the different body sites at the phylum level, all were dominated by Firmicutes and Bacteroidetes, followed by Proteobacteria, Cyanobacteria, Campylobacterota, and Actinobacteria (**Figure 4**), with the relative abundance percentage for the top five phyla listed in **Table 1**. Firmicutes showed significant differences in relative abundance between BPH/5 and C57 oral cavities (p= 0.002). Within the BPH/5 strain, Firmicutes exhibited a significantly decreased abundance in the placenta (31.3%) compared to the fecal (51.4%; p =0.002) and oral sites (54.2%; p=0.002). Within the C57 strain, the oral cavity displayed significantly higher Firmicutes abundance (91.8%) compared to the placental site (37.9%; p <0.001) (**Figure 5A**).

**Table 1:** Top five most abundant bacteria at the phylum level in each body site.

	Relative abundance (%)	
	BPH/5	C57
Placenta	1. Firmicutes (31.3%) 2. Bacteroidetes (14.4%) 3. Cyanobacteria (12.2%) 4. Proteobacteria (11.2%) 5. Actinobacteria (5.9%) Unknown (19.5%)	1. Firmicutes (37.9%) 2. Bacteroidetes (21.2%) 3. Proteobacteria (4.1%) 4. Actinobacteria (2.9%) 5. Cyanobacteria (1.7%) Unknown (30.5%)
Vagina	1. Firmicutes (27.9%) 2. Proteobacteria (21.5%) 3. Bacteroidetes (12.2%) 4. Actinobacteria (10.3%) 5. Cyanobacteria (6.4%) Unknown (16.6%)	1. Firmicutes (41.2%) 2. Proteobacteria (16.1%) 3. Bacteroidetes (12.8%) 4. Cyanobacteria (9.9%) 5. Actinobacteria (2.4%) Unknown (17.1%)
Oral	1. Firmicutes (54.2%) 2. Proteobacteria (15.4%) 3. Bacteroidetes (7.6%) 4. Campylobacterota (1.9%) 5. Cyanobacteria (1.4%) Unknown (18.3%)	1. Firmicutes (91.8%) 2. Proteobacteria (1.7%) 3. Bacteroidetes (0.8%) 4. Actinobacteria (0.6%) 5. Cyanobacteria (0.02%) Unknown (5.1%)
Feces	1. Firmicutes (51.4%) 2. Bacteroidetes (36.0%) 3. Campylobacterota (7.4%) 4. Cyanobacteria (2.5%) 5. Proteobacteria (1.1%) Unknown (0.6%)	1. Firmicutes (52.3%) 2. Bacteroidetes (40.6%) 3. Proteobacteria (3.9%) 4. Tenericutes (2.2%) 5. Actinobacteria (0.5%) Unknown (0.3%)

In contrast, Bacteroidetes did not show significant differences between BPH/5 and C57 across body sites (p > 0.05). Within the BPH/5 strain, the oral cavity exhibited significantly lower Bacteroidetes abundance (7.6%) compared to the placenta (14.4%; p = 0.014), while the fecal samples showed significantly higher abundance compared to the placenta (36%; p < 0.001). Similarly, within the C57 strain, the oral cavity showed significantly lower Bacteroidetes abundance (0.8%) compared to the placenta (21.2%; p < 0.001), and the fecal samples displayed significantly higher Bacteroidetes abundance compared to the placenta (40.6%; p = 0.0485) (**Figure 5B**).

Proteobacteria showed significant differences between BPH/5 and C57 at the oral sites (15.4 vs 1.7%; p = 0.017). Within the BPH/5 strain, fecal samples exhibited significantly lower Proteobacteria abundance (1.1%) compared to the placenta (11.2%; p < 0.001). In the C57 strain, no significant differences were detected between the placenta and other sites (**Figure 5C**). Cyanobacteria showed significant differences between BPH/5 and C57 at the placental (p = 0.008) and fecal sites (p = 0.029). Specifically, Cyanobacteria abundance was significantly higher in BPH/5 fecal (2.5%) and placenta (12.2%) compared to C57 controls (1.7%). Within the BPH/5 strain, the placenta showed significantly higher Cyanobacteria abundance compared to the oral cavity (1.4%; p = 0.005). In the C57 strain, the placenta showed significantly lower Cyanobacteria abundance (1.7%) compared to the vaginal cavity (9.9%; p = 0.016) (**Figure 5D**).

Campylobacterota (formerly Epsilonbacteraeota) differed significantly between BPH/5 and C57 at the placental (p = 0.017), vaginal (p = 0.003), and fecal sites (p < 0.001). Within the BPH/5 strain, fecal Campylobacterota abundance (7.4%) was significantly higher compared to placental site (p = 0.015), while no significant differences were detected between the placenta and other sites in the C57 strain (p > 0.05) (**Figure 5E**). Actinobacteria showed no significant differences by body site between BPH/5 and C57 (p > 0.05). Within BPH/5, the placental site exhibited significantly higher Actinobacteria abundance (5.9%) compared to fecal (p < 0.001) and oral sites (p = 0.027), while no significant differences were detected between the placenta and other sites in the C57 strain (p > 0.05) (**Figure 5F**).

### Relative abundance changes found at the genus level among body sites

**Figure 6:**
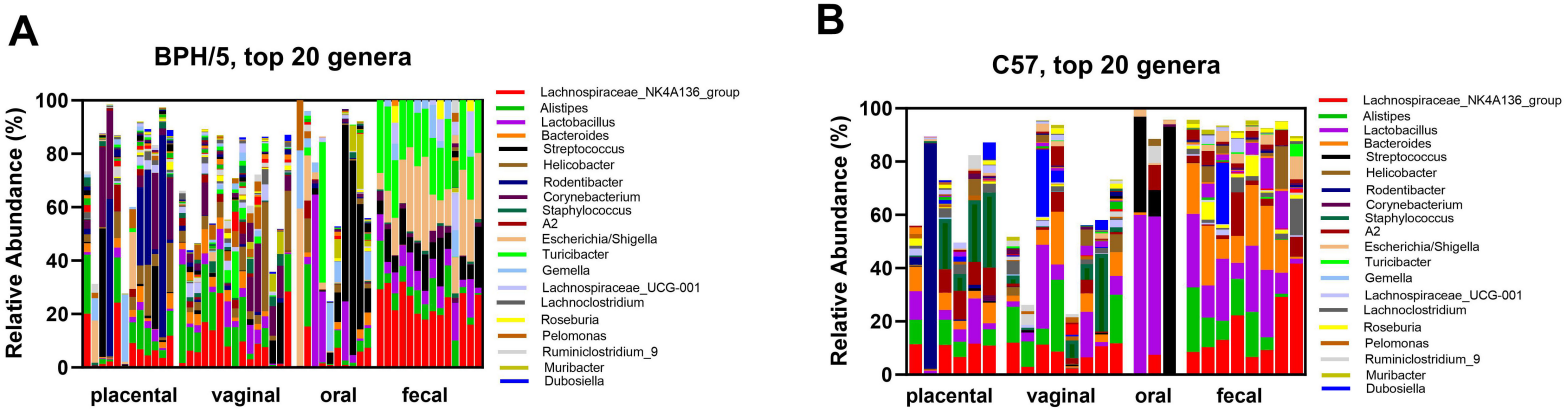
Relative abundance of the top 20 bacterial genera across body sites in BPH/5 and C57 mice. Stacked bar plots show the relative abundance (%) of the top 20 most abundant bacterial genera identified in the placental, vaginal, oral, and fecal microbiota of pregnant BPH/5 **(A)** and C57 **(B)** mice. Taxonomic classification was based on 16S rRNA amplicon sequencing. Each bar represents an individual sample. Sample sizes: BPH/5: fecal (n = 14), oral (n = 10), placental (n = 15), vaginal (n = 12); C57: fecal (n = 8), oral (n = 3), placental (n = 8), vaginal (n = 6).

**Figure 7:**
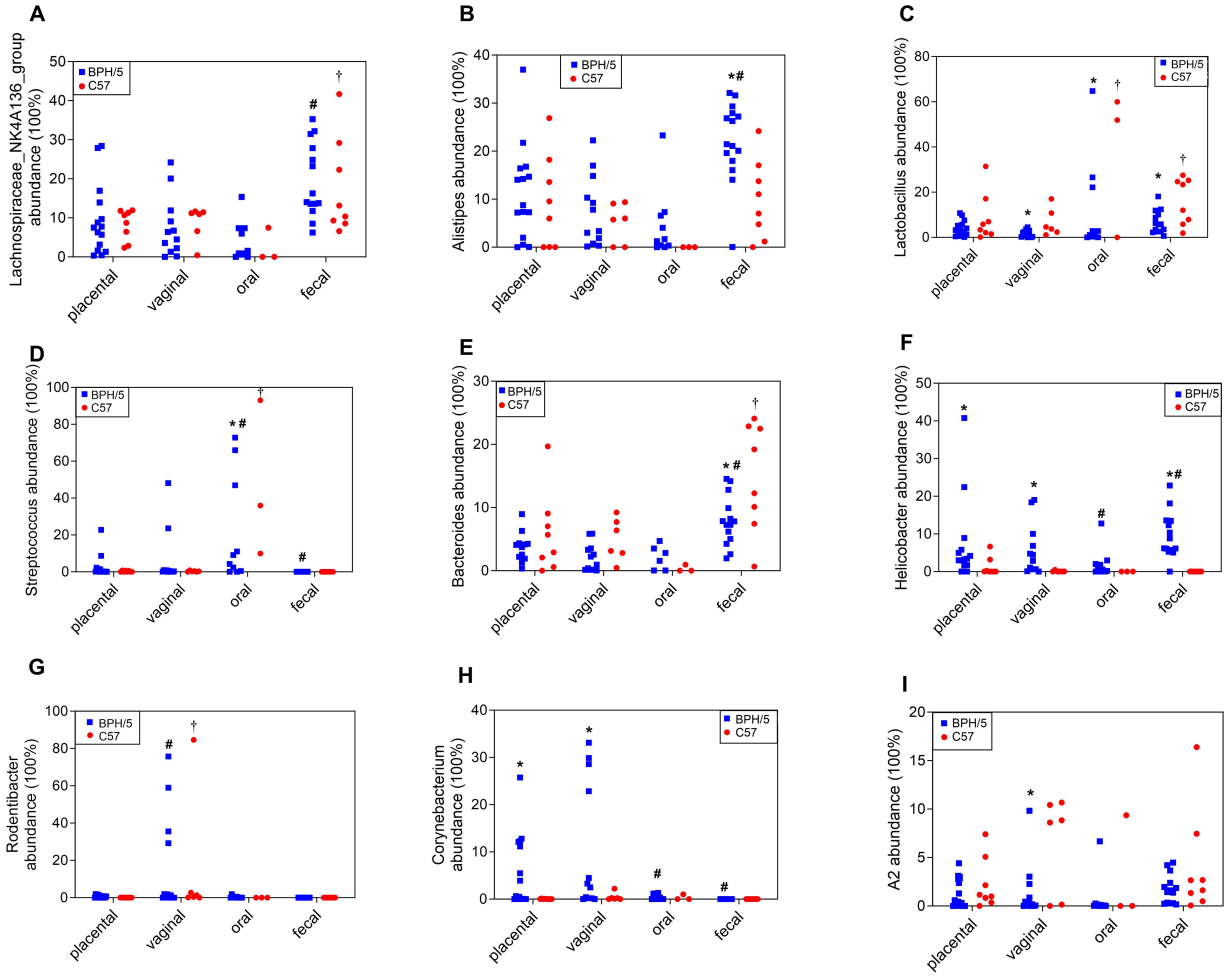
Relative abundance of selected bacterial genera in BPH/5 and C57 mice across four body sites. Relative abundance of selected bacterial genera in placental, vaginal, oral, and fecal samples from pregnant BPH/5 (blue squares) and C57 (red circles) mice: **(A)** *Lachnospiraceae_NK4A136_group*, **(B)** *Alistipes*, **(C)** *Lactobacillus*, **(D)** *Streptococcus*, **(E)** *Bacteroides*, **(F)** *Helicobacter*, **(G)** *Rodentibacter*, **(H)** *Corynebacterium*, and **(I)** *A2*. Statistical analysis was conducted using mixed ANOVA followed by *post hoc* pairwise LSD comparisons. (*p < 0.05 vs. C57 strain at the same site; †*p* < 0.05 vs. C57 placenta; #*p* < 0.05 vs. BPH/5 placenta). Sample sizes: BPH/5: fecal (n = 14), oral (n = 10), placental (n = 15), vaginal (n = 12); C57: fecal (n = 8), oral (n = 3), placental (n = 8), vaginal (n = 6).

When evaluating the different body sites at the genus level, prominent genera emerged in both BPH/5 and C57 strains, including *Lachnospiraceae_NK4A136_group*, *Alistipes, Lactobacillus, Streptococcus, Bacteroides, Helicobacter, Romboutsia, Corynebacterium*, and *A2* (**Figures 6A, B**). *Lachnospiraceae_NK4A136_group* showed no significant differences in relative abundance between BPH/5 and C57 across all four sites (p > 0.05). Within both the BPH/5 and C57 strains, fecal samples exhibited significantly higher abundance compared to the placenta (p < 0.001 and p = 0.013, respectively) (**Figure 7A**).

*Alistipes* showed significant differences between BPH/5 and C57 strains in fecal samples (p = 0.001). Within the BPH/5 strain, fecal samples showed significantly higher abundance compared to the placenta (p < 0.001). No significant differences were observed between the placenta and other sites in the C57 strain (p > 0.05) (**Figure 7B**). *Lactobacillus* displayed significant differences between BPH/5 and C57 strains in vaginal (p = 0.044), oral (p = 0.002), and fecal samples (p = 0.048). Within the BPH/5 strain, no significant differences were detected between the placenta and other sites (p > 0.05). In contrast, the C57 strain exhibited increased abundance in both oral (p < 0.001) and fecal sites (p = 0.029) relative to the placenta (**Figure 7C**). Relative abundance of *Streptococcus* showed significant differences between strains in the oral site (p = 0.014). Within the BPH/5 strain, the oral site had significantly higher abundance (p < 0.001), and the fecal site had significantly lower abundance (p = 0.044) compared to the placenta. In the C57 strain, oral samples exhibited elevated levels relative to the placenta (p < 0.001) (**Figure 7D**).

*Bacteroides* displayed significant differences between strains in the fecal site (p < 0.001). Within both C57 and BPH/5 strains, fecal samples showed significantly higher abundance compared to the placenta (p = 0.002 and p = 0.030, respectively) (**Figure 7E**). *Helicobacter* exhibited significant differences in relative abundance between BPH/5 and C57 strains across placental, vaginal, and fecal samples (p = 0.006, 0.002, and <0.001, respectively). Within the BPH/5 strain, oral samples showed significantly lower abundance (p = 0.022), and fecal samples exhibited significantly higher abundance (p = 0.025) compared to the placenta (**Figure 7F**).

*Rodentibacter* showed no significant differences between strains across the four sites examined (p > 0.05). However, within both BPH/5 and C57 strains, vaginal samples demonstrated significantly higher abundance compared to the placenta (p < 0.001 and p = 0.019, respectively) (**Figure 7G**). *Corynebacterium* displayed significant differences between strains in placental and vaginal samples (p = 0.002 and p = 0.004, respectively). Within the BPH/5 strain, placental samples had significantly higher abundance compared to oral (p = 0.014) and fecal sites (p = 0.004) (**Figure 7H**). *A2* showed significant differences between BPH/5 and C57 strains in vaginal samples (p = 0.011). No significant differences were observed between the placenta and other sites within both strains (p > 0.05) (**Figure 7I**).

### The estimated placental microbial signatures have many potential contributing sources

**Figure 8:**
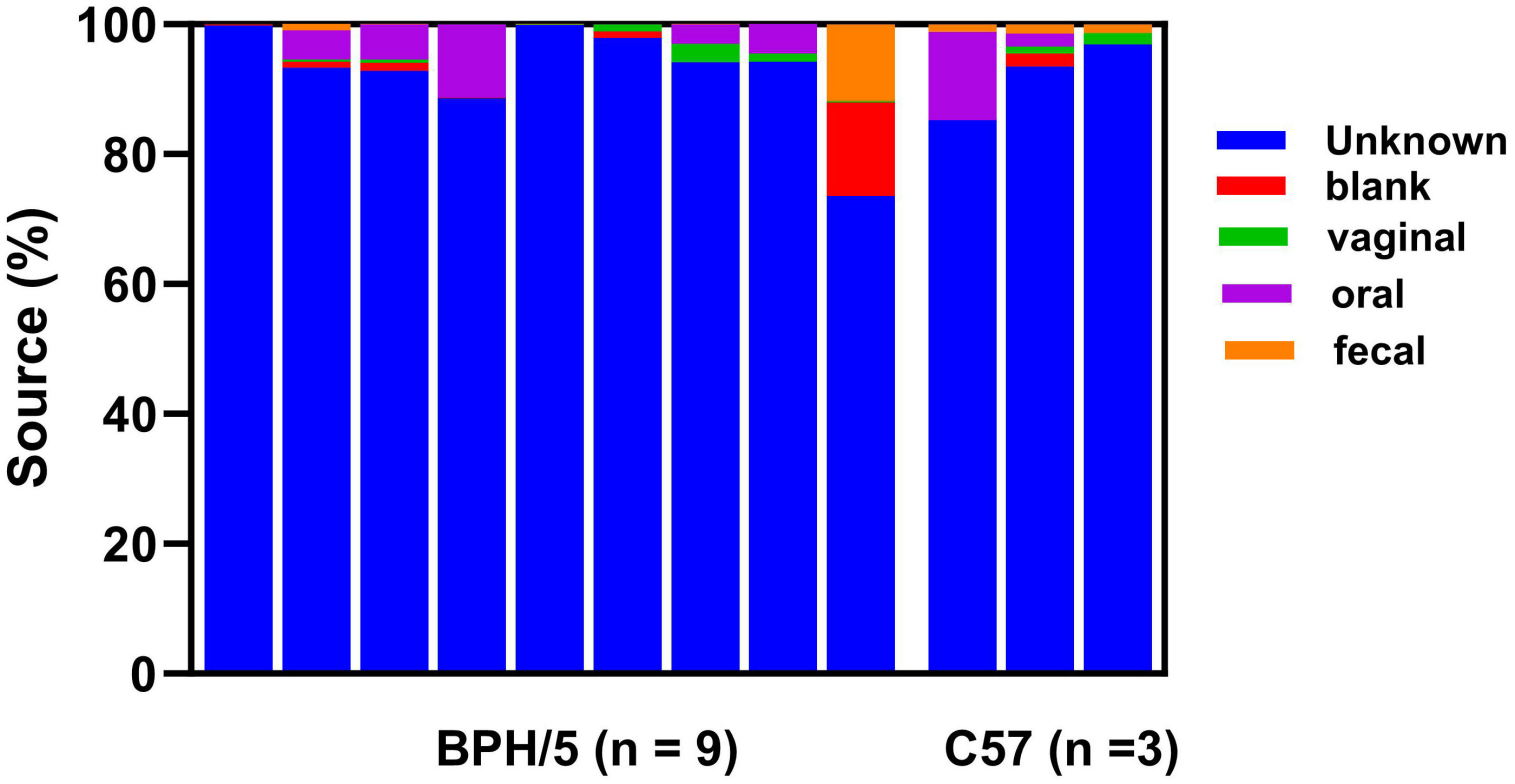
Microbial source tracking of placental samples from BPH/5 and C57 mice. SourceTracker2 analysis was used to estimate the proportion of microbial communities in placental samples originating from potential sources, including oral, vaginal, fecal, blank, and unknown sources. Results are shown for samples from pregnant BPH/5 (n = 9) and C57 (n = 3) mice, with all four source sites included.

Microbial communities consist of multiple sources, including potential contaminants from bacterial communities, sampling, or DNA extraction. In next-generation sequencing studies, distinguishing true microbial sources from environmental or iatrogenic contamination is essential. To address this, the SourceTracker2 package in Python was used to trace the origins of microbes found in placental samples. A total of 12 samples (BPH/5, n = 9; C57, n = 3) were analyzed across all four available body sites. Results varied by individual mouse, but in BPH/5 mice, the majority of the placental microbial signatures originated from an unidentified source, suggesting a unique microbial profile distinct from other maternal body sites. The oral cavity was the second-largest contributor, followed by the vagina. A similar trend was observed in C57 mice. Notably, the inclusion of blank controls confirmed minimal contamination, with blank contributions remaining at 2% or lower. In contrast, the oral cavity contributed between 1% and 14%, while the vaginal contribution was 2% or less. A particularly significant finding was that 85% to 99% of microbial contributions in placental samples remained classified as “unknown” (**Figure 8**). This highlights the need for further research to characterize these unidentified microbes and understand their potential role in pregnancy and placental health.

## Discussion

The overall goal of this study was to characterize the maternal microbiome from multiple body sites during late gestation in an obese hypertensive mouse model. Our hypothesis was that the pregnant obese preeclamptic-like BPH/5 mouse will demonstrate distinct placental microbial signatures that differs from the oral, fecal and vaginal microbiomes. Also, BPH/5 will have perturbed microbial communities compared to lean normotensive C57 controls. The main findings include 1) BPH/5 displays evidence of maternal microbial dysbiosis as bacterial communities of all body sites sampled were significantly different from normotensive lean controls despite the same environment and feeding regime, 2) When assessing beta diversity microbial community structure, the BPH/5 placenta was most similar to the vagina, while all other body sites were significantly different from one another, and 3) Overall the fecal microbiome was the most diverse community in both BPH/5 and C57, and grouped similarly within strain, followed by the placental, vaginal, and oral cavities. Determining the biological significance of these findings is ongoing. Noteworthy, the BPH/5 oral, placental, and vaginal cavities demonstrated a wide variety of individual variation. The BPH5 placenta was dominated by unclassified bacteria followed by *Alistipes, Lachnospiraceae_NK4A136_group, Helicobacter*, and *Corynebacterium.* Due to the difference in community structure, species richness, diversity, and relative abundance differences between body site and normotensive control strain, the placenta did harbor its own unique microbiome.

Previous studies have reported healthy human placental microbial signatures consisting of: Firmicutes, Tenericutes, Proteobacteria, Bacteroidetes, and Fusobacteria (Aagaard et al., 2014; Pelzer et al., 2017). More specifically, *Lachnospiraceae, Bacteroides*, and *Lactobacillus* have been found in human studies on the placental microbiome (Aagaard et al., 2014; Pelzer et al., 2017; Williams et al., 2022). Similarly, those genera have been isolated in both BPH/5 and C57 placentas in this study. In humans, the placenta was found to be most similar to the oral microbiome, with the oral microbiome being a major contributor to bacterial translocation and potential source of infection during pregnancy. Within the BPH/5 and C57 strains, the placenta was found to be significantly different from the oral cavity when assessing alpha and beta diversity but shared multiple taxa (including but not limited to *Lactobacillus, Streptococcus, Corynebacterium, Alistipes, Lachnospiraceae_NK4A136, and Helicobacter)*. Within this study based on diversity alone, the BPH/5 and C57 placenta most similarly grouped with the vaginal samples sharing multiple taxa and no differences using *post hoc* analysis in alpha or beta diversity. While using sourcetracker, the oral cavity showed the greatest potential contribution to the placenta in both strains, contributing up to 14% within certain samples. Highlighting the need for more research to determine the biological significance of the placental microbiome sources.

Revisiting the original question “How do microbial signatures reach the placenta?” The current proposed hypothesis is that placental colonization happens in three potential routes. First, vertical translocation from vagina (Goldenberg et al., 2008), secondly, hematogenous spread from the gut (Perez et al., 2007), and lastly, hematogenous spread from the oral cavity (Fardini et al., 2010). The vagina is an important aspect of maternal, fetal, and overall reproductive health. In humans, preterm birth has also been associated with vaginal microbiome instability. A suppression of the vaginal microbial richness and diversity during the first two trimesters resulting in preterm births may suggest that proper vaginal microbial colonization is needed to maintain a successful pregnancy (Stout et al., 2017). Within this study, the vaginal vertical translocation of select microbes seems applicable due to the similarity of the placental microbiome to the vaginal microbiome in both late gestation BPH/5 and C57 mice. Notably, *Lactobacillus* sp. was in the top five bacteria identified in the C57 late gestation vagina, while not in BPH/5. This was associated with 8.5% relative abundance of *Lactobacillus* spp. in the C57 placental microbial signatures and only 4% in BPH/5. The human uterine microbiome shares similar taxa from the phyla Firmicutes and Actinobacteria with the vaginal microbiome such as *Lactobacillus, Bacteroides, Gardnerella and Prevotella* (Verstraelen et al., 2016). The BPH/5 mouse shares both *Lactobacillus* and *Bacteroides* between the vagina and the placenta samples albeit at levels lower than C57. *Lactobacillus* is thought to play a major role in preventing overgrowth of pathogenic bacteria. Bacterial vaginosis is now characterized by a reduction in *Lactobacillus*, which would be a dysbiosis of the natural vaginal microbiome and could be detrimental to the pregnancy (Klebanoff et al., 1991). Even though it is possible that *Lactobacillus* in all three body sites plays a critical role in contributing to the developing placental microbiome as supported by its presence in the oral, vaginal, and fecal microbiomes of C57. Others have evidence to support the oral and gut microbiome contributing to the placental microbiome in overweight and obese women (Gomez-Arango et al., 2017); however, the pregnant vaginal microbiome was not measured. Preeclamptic infants have been found to lack *Lactobacillus* and are instead dominated by Proteobacteria (Barrett et al., 2013). More studies are needed to understand the role of maternal co-morbidities in the transmission of microbiomes to offspring in pregnancy.

The oral microbiome as a contributing source of the placental microbiome was identified in the current study by sourcetracker, but has previously been linked in humans, horses, and cows (Aagaard et al., 2014; Hummel et al., 2022; Beckers et al., 2025). In another rodent study, pregnant mice were challenged with *Porphyromonas gingivalis* (Bacteroidetes), a common oral pathogen. This led to decreased fetal weights, increase fetal reabsorption, and fetal growth restriction (Collins et al., 1994). Although not supported by this study, the microbiome of the placenta has been described to be more similar to that of the oral cavity, it is hypothesized that oral pathogenic microbes that disseminate hematogenously cause intrauterine infection when periodontal infection is present (Bearfield et al., 2002). Additional rodent models have also been used to study the translocation of bacteria to the placenta from the oral cavity (Fardini et al., 2010) as well as intestinal-derived labeled bacteria into the placenta (Jiménez et al., 2008). Challenging the pregnant mice with *Campylobacter rectus* (Proteobacteria) resulted in abnormal placental architecture and inflammation and increased pup mortality (Offenbacher et al., 2005). The oral microbiome has also been linked to the development of PE and preterm births (Cobb et al., 2017). Oral dysbiosis caused by dental caries, gingivitis, and periodontitis have been linked to cause inflammatory immune responses that lead to adverse pregnancy outcomes (Boggess et al., 2003). A pertinent bacterium that has been found in multiple human studies to translocate from the oral cavity to the placenta is *Streptococcus (*Gomez-Arango et al., 2017). Our study also demonstrates S*treptococcus* DNA being found in high abundances within the oral cavity of C57 (46.3%) and BPH/5 (21.3%) as well as the vaginal cavity of BPH/5 but not C57 concordant with a lower abundance within placentae. In humans, it was also shown that women positive for *Streptococcus* in their placenta and their oral cavity had a significantly higher prevalence of PE (Cooper et al., 2024). Which is a similar finding in our study where the *Streptococcus* in the preeclamptic BPH/5 placenta is 10-fold higher than pregnant C57 controls. SourceTracker-based microbial source attribution should be interpreted cautiously, particularly in low-biomass tissues where limited microbial signal and incomplete reference databases may result in a high proportion of unassigned or unknown source contributions. Therefore, the predicted origins of placental microbial signatures in this study represent potential associations rather than definitive evidence of microbial transmission or colonization, and future studies incorporating expanded reference communities and orthogonal validation methods will be necessary to further define microbial sources.

The microbiome of the pregnant BPH/5 mouse differs from the normotensive C57 microbiome potentially indicating a level of dysbiosis that may contribute to systemic inflammation described in this model. The BPH/5 model has been shown to demonstrate increased inflammation in the placenta, WAT, and colon which could be a result of a dysbiotic microbiome (Sutton et al., 2017; Reijnders et al., 2019; Beckers et al., 2025). Specifically, increased interleukin (IL)-15, a pro-inflammatory cytokine in the adipose tissues and implantation sites as previously described (Reijnders et al., 2019). The placenta plays a critical role in PE pregnancies; some suggest that placental microbial dysbiosis is associated with the development of PE. Pathogenic bacteria such as *Bacillus cereus* (Firmicutes), *Listeria* (Firmicutes), *Salmonella* (Proteobacteria), *Escherichia* (Proteobacteria), *Klebsiella pneumonia* (Proteobacteria)*, Anoxybacillus* (Firmicutes), *Variovorax* (Proteobacteria), *Prevotella* (Bacteroidetes), *Porphyromonas* (Bacteroidetes), and *Dialister* (Firmicutes) were found in seven placental samples from mothers with PE (Barak et al., 2007; Amarasekara et al., 2015). Another study by Kell and Kenny found that dormant microbial infection that overgrow in abundance are responsible for chronic inflammation and PE sequelae (Verstraelen et al., 2016). Within our BPH/5 mouse model in late gestation, there is an overabundance of placental *Helicobacter* likely originating from the GI tract, *Streptococcus* potentially from the oral cavity, and *Corynebacterium* detected within the vagina. This may contribute to increased systemic inflammation and thus the cascade associated with PE within this model. Furthermore, this supports the pathogenic infection theory that a single infectious event could trigger an inflammatory response with pro-inflammatory cytokines and complement proteins that have been implicated in PE-specific abnormal placentation (Lynch et al., 2008; Regal et al., 2017; Olson et al., 2019).

Another aspect of the BPH/5 model is spontaneous obesity. In humans, the obese maternal placenta has been shown to be dominated by Firmicutes and Proteobacteria phyla (80% of total microbial abundance) (Gomez-Arango et al., 2017). The most dominant organisms found in the phylum Firmicutes included *Streptococcus, Lactobacillus*, and *Veillonela. Pseudomonas, Haemophilus* and *Acinetobacter* dominated the phylum Proteobacteria (Gomez-Arango et al., 2017)*. S*imilar to C57 and BPH/5 pregnant mice, was the presence of *Streptococcus* and *Lactobacillus* within the gut (Luo et al., 2017). Recently, the early gestation fecal microbiome in BPH/5 revealed 6.1% relative abundance of *Lactobacillus (*Fan et al., 2026) that was found in this study to increase only to 6.7% in late gestation while C57 increased almost three-fold with pregnancy. This suggests *Lactobacillus* has a PE biomarker in the BPH/5 model. The potential method of translocation from the gut during pregnancy possibly from leaky gut syndromes which commonly develops during pregnancy (Koren et al., 2012; Beckers and Sones, 2020) warrants further exploration in this model.

This study faces several limitations, primarily the debate over whether a placental microbiome exists. Recent research, particularly in humans, challenges this notion. Lauder et al. used PCR and Illumina sequencing with contamination controls and found that placental samples contained low and indistinguishable 16S rRNA copy numbers compared to extraction blanks (Lauder et al., 2016). Similarly, PERMANOVA analysis of Bray–Curtis and UniFrac distances showed no community separation. Perez-Muñoz et al. argued that current next-generation sequencing methods are flawed due to low biomass, making bacterial DNA detection unreliable and highly susceptible to contamination (Perez-Muñoz et al., 2017). Leiby et al. further supported this concept, finding no distinction between background negative controls and placental samples in cases of spontaneous preterm birth (Leiby et al., 2018). These data suggest that the placenta may be sterile until membrane rupture during delivery with microbiome detections in cesarean sections likely resulting from contamination and often from DNA extraction kits (Lauder et al., 2016). It is necessary to examine every step of the sequencing process to assess the accuracy of the core microbiome of the placenta. To address potential contamination, this study implemented stringent controls. Mice were humanely euthanized to prevent contamination from membrane rupture or birth. The uterus was aseptically removed *in situ*, swabs were sterilely collected, and samples were immediately frozen. Additionally, blanks were taken at every stage including environmental sampling, DNA extraction, and PCR then analyzed using the *decontam* package in R. None of the study samples matched the blanks, ensuring data integrity. Nonetheless, this study is descriptive and more studies are necessary to link maternal dysbiosis with inflammation and hypertension in this model. These findings should be interpreted cautiously and that future studies incorporating orthogonal validation approaches will be necessary to determine whether these microbial signatures represent viable microbial communities, transient microbial DNA, or other sources of bacterial DNA.

Studies identifying specific pathobionts behind gut microbial dysbiosis remain essential in understanding the etiology of PE. The advantage of the BPH/5 model is its spontaneous development of PE with obesity without surgical or pharmacological intervention, allowing longitudinal study of microbial changes without external influence. This model will be valuable for future investigations into pregnancy supplementation, i.e. probiotics, and their effects on maternal and fetal PE outcomes. The role of the maternal microbiome in PE remains largely unexplored, particularly the influence of microbiomes from multiple maternal body sites.

In conclusion, our findings in BPH/5 late gestation mice suggest a dysbiosis of the maternal microbiome during pregnancy and adverse outcomes. Early diagnosis of dysbiosis could facilitate interventions to mitigate PE risk. Probiotic therapies, particularly with *Lactobacilli*, may help prevent pathogenic overgrowth and maintain a healthy *in utero* environment. The exact timing of placental microbiome seeding remains unclear, but potential vertical transmission from the vagina or hematogenous spread from the gut or oral cavity suggests that maternal microbiome health at these sites may directly influence pregnancy outcomes.

## Data Availability

The original contributions presented in the study are publicly available. This data can be found here: NCBI Sequence Read Archive, accession number PRJNA1292389.
